# The perceived experience of adhering to vegan diet: a descriptive phenomenological study

**DOI:** 10.1186/s12889-024-18227-0

**Published:** 2024-03-11

**Authors:** Behnam Khaledi-Paveh, Alireza Abdi, Sousan Heydarpour, Fatemeh Dehghan, Reza Haghparast, Hooman Ghasemi

**Affiliations:** 1 Faculty, Department of Psychiatry Nursing, Faculty of Nursing and Midwifery, Kermanshah University of Medical Sciences, Kermanshah, Iran; 2 Department of Emergency and Critical Care Nursing, Faculty of Nursing and Midwifery, Kermanshah University of Medical Sciences, Kermanshah, Iran; 3 Department of Reproductive Health, Faculty of Nursing and Midwifery, Kermanshah University of Medical Sciences, Kermanshah, Iran; 4 Psychology, nursing, and midwifery school, Kermanshah University of Medical Sciences, Kermanshah, Iran; 5 Plant Science, Agriculture Research, Education and Extension Organization (AREEO), Dryland Agricultural Research Institute, Kermanshah, Iran; 6 Student Research Committee, Kermanshah University of Medical Sciences, Kermanshah, Iran

**Keywords:** Vegetarianism, Perceived experience, Phenomenology, Self-care, Vegan

## Abstract

**Background:**

Today, raw vegetarianism is considered one of the most important socio-cultural developments in contemporary societies. In this regard, the present study was conducted to describe the perceived experience of people adhering to a vegan diet through a phenomenological perspective.

**Methods:**

This qualitative study explores the perceived experiences of individuals who follow a vegan diet and are part of the self-care campaign in Kermanshah, Iran. sampling was purposeful and face-to-face interviews were conducted with 12 individuals who follow a vegan lifestyle. The data were analyzed after being collected using the seven steps of Collizi. MAXQUDA software (version 12) was used for data management.

**Results:**

After qualitative data analysis, we identified 567 codes, which were categorized into 15 sub-themes. From these sub-themes, we derived 4 main themes. The main themes include: In pursuit of redemption (Meaningful framework, In awareness path, Unequaled Disappointment, Chronic and complex conditions), Seeking the New World (Starting with doubt and hesitation, The temptation to return, Constant criticism, Unfamiliar appearance), On the path of overcoming difficulties (Associate problems, Matching the new way, Perceived recommendations), and attaining the expected outcomes (Healthy lifestyle, Therapeutic feedback, Enhancing emotional wellbeing, Feeling of youth).

**Conclusion:**

Based on the participants’ experience, despite the challenging journey, the people with a vegetarian diet experienced partial and complete improvement of all the symptoms of the diseases. They had a healthy lifestyle and felt young and energetic. Likewise, this method had a positive effect on people’s mental state and mood.

## Background

Today, non-communicable diseases (NCDs) such as cardiovascular disorders, diabetes, cancer, and respiratory diseases, account for over 70% of deaths, totaling 41 million people [1]. These disorders cause more than 8.2 million years of life with disability in Iranian people every year [2]. Some believe that the lack of control of NCDs in the coming years will result in the deaths of over 80% of people in various societies and will impose the greatest burden of diseases on mankind. In response, the World Health Organization has developed a program to address this epidemic, focusing on changing health-related policies regarding lifestyle modifications [1–3].

NCDs occur due to various causes, such as unhealthy diet, lack of sports activity, and consumption of smoking substances and alcohol, which ultimately lead to obesity, diabetes, and other disorders [4, 5]. The 2016 research on the average body mass index from 1975 to 2014 revealed an increase from 21.7 to 24.2 kg/m2 for men, and from 22.1 to 24.4 kg/m2 for women, leading to numerous non-communicable diseases [6].

Diet is known as the most important cause of NCDs [7]. Based on the 2019 Global Burden of Disease study, an unhealthy diet is a significant contributor to the burden of diseases [8]. The reduction of consumption of fruits and vegetables in developing countries and the increase in salt, fat, and canned foods consumption in developed societies have been noted as risk factors for NCDs [9]. In Iran, during the last 20 years, there have been important changes in the diet, and the consumption of animal proteins, and canned and prepared foods have risen [10].

The vegetarian diet is considered one of the new approaches of this century to prevent NCDs [11]. The reason for their effect is that vegetables and fruits are rich in substances such as antioxidants, fiber, vitamin C, and folic acid [12]. The phenomenon of vegetarianism has been considered not only as a way to eat but also as a philosophy and a way to live [13]. The vegetarian attitude is based on non-violence towards living beings and abstaining from meat and other animal products [14]. A meta-analysis study in 2018 shows that a vegetarian diet has reduced the average body mass index of people by 1.49 kg/m2, and has reduced cholesterol (28.16 mg/dL), LDL (21.27 mg per deciliter), triglyceride (11.39 mg/dL) and blood sugar (5.08 mg/dL), and also reduced the prevalence of various cancers by 8–10% [15].

Although the positive effects of vegetarian diets have been quantitatively confirmed in various studies [15–17], Considering the presence of negative attitudes and cultural/religious issues in various societies, such as the decrease in male sexual characteristics [18], the difficulty in changing dietary habits, the preference for consuming meat products [19], and insufficient intake of essential nutrients such as vitamin B12 and calcium; Compliance with these dietary choices has not been widely addressed, resulting in a reported less prevalence of individuals opting for a vegetarian lifestyle [20].

According to the Health Belief Model, studying the experiences and attitudes of those involved is necessary to assess adherence to health behavior. This suggestion also applies to the vegetarian culture [19]. A qualitative study revealed that individuals encounter numerous practical and social challenges when adhering to plant-based or vegetarian diets. One of the key themes identified in the study was the participants’ concerns regarding social acceptance and acceptance within their community and family. Additionally, the participants expressed their desire to switch to a vegetarian diet due to motives, expectations, and cues [21]. Another mixed-method study discovered that social and motivational factors significantly impact adherence to diets in diverse ways [22]. Nevertheless, further research is required to identify the motivational and social influences that impact the practice of vegetarianism.

To gain a deeper understanding of the phenomena, it is crucial to examine individuals with direct firsthand experience. Qualitative research, particularly employing the constructivist approach, has been utilized to delve into the lived experiences of individuals. These approaches maintain that the essence of human phenomena is best explored within lived experiences, with the researcher serving as a tool for study [23]. Given that adherence to specific regimens is influenced by culture, experience, and attitude [24], and that the researchers of this study are investigating vegan individuals and their health concerns in the Kermanshah self-care campaign. Therefore, the present study was conducted to describe the perceived experience of people adhering to a vegan diet through a descriptive phenomenological qualitative study.

## Methods

This study was a descriptive phenomenology (Husserl’s phenomenology) under the philosophy naturalistic or constructivist (23), conducted on individuals introduced by Kermanshah City’s self-care campaign who experienced vegetarianism and were willing to participate. In order to recruit participants, after taking the participant’s cell phone numbers from the campaign’s manager, contact was initially made by phone. for the invitation, and interviews were conducted at the first author`s office in the sleep research center of KUMS, where there is a friendly and calm environment. The participants were provided written informed consent, and the sampling method was purposeful and continued until data saturation was achieved; Ensuring that no new theme/subtheme emerged as data gathering continued. For data collection, a demographic information checklist including age, sex, history of vegetarianism, place of residence, underlying disease, and occupation of the participants was employed. Additionally, we conducted face-to-face semi-structured in-depth interviews using various open-ended questions e.g. “How has your experience of vegetarianism been?” and “How has vegetarianism affected your life?” We also used probing questions such as “Why?“, “How?“, and “Explain more”. The first and second author collected the data from October to February 2019. Finally, the interviews reached saturation in interview twelve. The interviews lasted between 20 and 45 min. After obtaining the written informed consent, the interviews were recorded on audio tape by Samsung cellphone. MAXQDA 12 software was used for managing the data. In this qualitative study, data analysis was done through the seven-step method of Collaizi which was noted in Shosha (2012) [25]. Likewise, at the end of each interview and recording, the audio file was listened to several times, and then we wrote it verbatim.

To understand participant perception, we thoroughly reviewed the interviews multiple times. Relevant information and statements related to the phenomenon of interest were highlighted. A line-by-line analysis of the participant’s descriptions was conducted to identify significant sentences and extract important notions/codes. The texted thoughts were carefully evaluated, and commonalities were classified according to the fourth step. Thematic portions were constructed from the written texts. In the fifth phase, outcomes were connected for a comprehensive research description, and further categories were developed. In the sixth step, a clear and unobtrusive description of the subject was provided. The last step of rigor was accomplished.

Overall, the Schwandt et al. technique [26] was used to ensure the trustworthiness of the data in this study. In order to have credibility, the researcher was engaged for about one year in the study, and peer debriefing and member checks were conducted. Dependability was achieved through a combination of data collection methods, and then the data was verified by an impartial observer. To assess the confirmability of the research protocol, an independent researcher reviewed the coding description, main themes, narrated narratives, and the capacity to track the narratives and articulate the themes. Eventually, the study assessed transferability by examining patient characteristics, sampling techniques, data gathering, and analysis methods.

The research followed ethical guidelines, including obtaining informed consent, maintaining confidentiality, and allowing participants to withdraw at any time. It was approved by the Kermanshah University of Medical Sciences Ethics Committee (code IR.KUMS.REC.1398.119).

## Findings

In this study, twelve people participated. Eight of them were female and the average age and history of a vegetarian diet was 25.50 years and 29.08 months, respectively. Most people went on a vegetarian diet due to illness, and the most common disorder was obesity. Table 1 shows the characteristics of the participants.

**Table 1:** Demographic characteristics of the participants (P)

Number	Age (year)	gender	Vegan duration	Diseases healed
P1	42	Male	1 year	Cancer symptoms, sleep, morality, stress
P2	53	Female	2 year	Colitis, Gastrointestinal ulcer
P3	53	Female	2 year	Colon cancer, GI bleeding, High blood pressure
P4	61	Female	5 year	Arthritic rheumatism
P5	42	Male	3.5 year	Obesity, restless leg syndrome
P6	52	Male	2 years and 2 months	Fatty liver degree 3, diabetes
P7	62	Female	7 Years	Breast Cancer
P8	40	Female	3 years	Arthritic rheumatism
P9	45	Female	1 year, 5 months	Obesity
P10	50	Female	3 months	Breast cancer
P11	58	Female	3 months	Obesity, knee pain, Heel spur
P12	45	Male	1.5 years	Obesity

After a qualitative analysis of the data, 567 codes were obtained, of which four themes and 15 sub-themes were extracted. The main themes included; In pursuit of redemption (Meaningful framework, In awareness path, Unequaled Disappointment, Chronic and complex conditions), Seeking the New World (Starting with doubt and hesitation, The temptation to return, Constant criticism, Unfamiliar appearance), On the path of overcoming difficulties (Associate problems, Matching the new way, Perceived recommendations), and Attaining the expected outcomes (Healthy lifestyle, Therapeutic feedback, Enhancing emotional wellbeing, Feeling of youth) (Table 2).

**Table 2:** Themes and subthemes after qualitative data analysis

Themes	Subthemes
In pursuit of redemption	Meaningful framework In awareness path Unequaled Disappointment Chronic and complex conditions
Seeking the New World	Starting with doubt and hesitation The temptation to return Constant criticism Unfamiliar appearance
On the path of overcoming difficulties	Associate problems Matching the new way’ Perceived recommendations
Attaining the expected outcomes	Healthy lifestyle Therapeutic feedback Enhancing emotional wellbeing Feeling of youth

### In pursuit of redemption

Participants in the research began the procedure for a variety of reasons, which are classified as sub-themes, Meaningful framework, In awareness path, unequaled disappointment, and Chronic and complex conditions.

#### Meaningful framework

Before adopting veganism, individuals identified several important principles that led to their adoption of vegetarianism. Most participants felt that this dietary choice was aligned with human nature and that our ancestors had previously thrived on it for long, healthy lives. In the past, humans lived like raw vegetarian animals, and even God paid attention to their diet. While some may believe that meat is the only source of protein, soybeans contain significantly more.

Four participants experienced the start of their vegan lifestyle as a spiritual matter and called it a miracle, believing it was God’s will. The seventh participant thanked God for the illnesses that led him to this diet, saying, “Thank God for these 7 years. I am very well and many times in my nature I thanked God for my problems. I also now say that the raw plant has not healed the disease, but God has cured and everything is in God’s hand.”

The participants found that they became more aware of their bodies and how they functioned based on their experiences, and they argued for addressing the root cause of the illness rather than just treating it. " I found hope and certainty on my path and learned what was good for me and what was damaging. I blame myself for any hardships and always examine what I did and ate when my blood sugar rises. I investigated the source and never made the same mistake again” (participant 5).

#### In awareness path

Newcomers to this particular dietary regimen sought to expand their understanding. Participant Number Twelve advised that the initial approach to overcoming challenges is to “boost your awareness to a level where you can rationally justify everything and have a scientific foundation for identifying problematic foods. Science-backed evidence is more convincing and trustworthy to him.”

These people explained it with research that approved of the features and benefits of vegetable foods and recommended it to others. “The apple tree works miracles and swiftly flushes, chili and water for breakfast, vegetable water for individuals with liver problems, salad for lunch, and medicinal herbs are also good for bleeding. Carrot juice and celery juice are excellent, Pomegranate and parsley juice is also good, salad dinner is excellent, vegetable raw sweets are excellent, strawberries and their solvents are very useful, utilize the diet channels and aid, this diet is beneficial for many conditions” (Participant 1).

Some of the participants saw vegetarianism as a spiritual refinement that could aid their physical recovery, and they learned about their peers’ experiences; “I was taking 18 tablets every day… I advised you to repair your soul, not backbite, deceive, or judge, and to have trust that God can heal cancer” (Participant 7).

#### Unequaled disappointment

Participants had come across a sort of disappointment before the vegetarian began that they had not experienced before. This disappointment was manifested in the form of dissatisfaction with life, becoming depressed, and waiting for death. Participant number nine, who was obese, explained his mental condition before vegan; “my weight had reached 138 kilograms due to pregnancy and afterward did not decrease at all, I was very ill and depressed, and I did not enjoy having a girl at all… I had lost my passion for life, and I had no pleasure in anything I was waiting for when I would die”.

Regarding having symptoms of depression, Participant Number 3 explained; “before the raw vegetable, my friends brought me a carpet panel to relieve my depression but I was so numb that I couldn’t tie and weave (the carpet), I couldn’t go to the kitchen and bring a glass of water and take my pills”. Some participants count killing animals for human food as disappointing and suffering work.

#### Chronic and complex conditions

Most of the participants had progressive diseases for which they had experienced several treatments, such as rheumatoid arthritis, diabetes, cancer, morbid obesity, etc. However, despite the treatments received, the result was not satisfactory to their point of view and they were looking for a more efficient and less complicated solution. Participant No. 4 stated: “I had bone pains about 6 and 7 years ago, it gradually got worse and I faced it throughout the day. I went to the orthopedic doctor because I wasn’t aware that it had nothing to do with orthopedics so they examined me and gave me some medications and told me that this pain will be with you for the rest of your life and you have to tolerate it”.

Participant number six had multiple illnesses and declared that the treatments he received made his condition worse. “In 1997, I went to more specialists and they said that polyps had started, my (Blood) sugar was rising, and my fatty liver was rising, 3 years ago I had grade 3 and I was afraid of liver cancer, and two ulcers were seen in my stomach, and I was diagnosed with stomach cancer because my relatives had taken it, I was scared, I went to a nutritionist and he gave me a diet plan, and because of that I was getting fatter and added to my problems”.

Participant number 3, who was diagnosed with gastrointestinal cancer, also articulated,” I went to the specialists and the doctor diagnosed malignant colon cancer and said that you should undergo chemotherapy and surgery and put a bag (colostomy bag), but because my father also had this problem and I saw how much he bothered, I stopped going for treatment because I was afraid… I couldn’t sleep because of fear. I was in severe pain and had to take morphine to sleep for a couple of hours”.

These progressive diseases and the treatments that most of the participants were faced with, made them try new methods such as vegetarianism. “I was invited to the forum and for a few days I got information and read the book of the deceased Avansian (the father of vegans in Iran) and I chose this path knowingly” (participant 5).

### Seeking the new world

Participants initially enter the vegetarian diet with skepticism, although they are tempted to return and sometimes make mistakes/errors (they call it for eating meat and baked food). Due to the change in their appearance, they are constantly criticized by those around them. For the experience of the vegetarian world, four sub-themes were obtained.

#### Starting with doubt and hesitation

Some participants did not accept the effectiveness of the vegetarian diet at first and entered this process because of curiosity and medical problems. But when people started to recover, they adhered to this method and got motivated to continue the path.

Participant number 9, who suffers from severe obesity, stated; “Everyone should accept this nutrition method wholeheartedly and then enter it. I didn’t start with it wholeheartedly at first, but then I fell in love with it. I now recommend to those who want to start to continue this method for 40 days and Don’t slip and assume that he is fasting”.

Participant number 5 had tried different ways to treat his disease and stated about the beginning of raw vegetarianism; “My other problem was hereditary diabetes and my breakfast sugar (fasting blood sugar) was always 130. I was pre-diabetic and I was very sick. Until I met Dr. … and Dr.… (the officials of the campaign) and was invited to the forum and spent a few days getting information and reading the late Avansian’s book and knowingly chose this path, I said that I had gone all the way and leave this way. I also tried and became a raw vegetarian”.

#### The temptation to return

The temptation to return to a previous diet was one of the most difficult conditions faced by participants at the start of the vegetarian path, and almost all participants mentioned it. The smell of food, attending special events, and seeing other people consume these types of foods were tempting factors to return. Also, in some cases, these participants made so-called errors and ate a previous diet, which in some cases caused a recurrence of symptoms. Participant number 1 stated, “The first months the smell of the food bothers me and it is tempting… When I started the diet (vegetarian diet) it was two days before Eid al-Adha (one of the eves for Muslim people). I used to sacrifice two sheep and I was addicted to red meat, but this time even the smell of its smoke bothered me (I didn`t eat)”.

Participant No. Two also had this in mind, saying that he was tempted to see the foods he had previously liked. “If I want to slip and be tempted, it’s very agonizing, and it’s annoying. I was tempted to see Abgosht (an Iranian food), I like the Ghorme Sabzi (an Iranian food) but I didn’t even allow myself to taste it because I have suffered so much from my illness and I do not want it to happen again. I promised myself that even if I had died not to eat any cooked food, no meat or barbecue, I would have noticed a rapid change in pinch/stint on food. Until I want, my disease will be stopped”.

#### Constant criticism

By entering vegetarianism, the participants were subjected to constant criticism from the people around them and their families, which made them feel isolated. In some cases, the participants were even accused of having a specific disease or drug addiction. Participant No. 7 stated: “When I lost weight, my family opposed me and said, ‘Eat to live’ and now the others say that she died of starvation and has nothing to eat, it was my younger brother’s wedding that my relatives had seen (me, and) said that she went to Tehran and it is not clear what she has done to himself, would be drug abuser, and it is better to take care of her until she is well…I tried to convince them and I preferred to answer them with a word, when I eat meat, I would have hives”.

Participant No. 6 also declared about the family’s advice: “There was a lot of negativity in my family and people around me, both my mother and my family were saying why you don’t eat, what trouble are you bringing on yourself, unless vegetables and fruits should be eaten (would be eaten instead of food)”.

In this regard, participants had to respond by making excuses, but after a while and seeing the effective results of the diet, criticism was reduced and other family members tended to be vegetarian.

#### Unfamiliar appearance

At the beginning of the vegan diet, participants experienced multiple physical changes, such as severe weight loss, hair loss, and changes in facial appearance. Both the participants and those around them considered these symptoms as a sign of weakness and illness. However, some participants, like Participant Number 1, experienced the drastic physical changes as a sign of spiritual awakening and tried to continue the path. Participant number 4 stated: “I experienced severe hair loss after 2 years on this diet, and it was very intense. When I talked to one of the raw vegan teachers, they gave me some methods to improve, and my hair turned grow. They said the reason is that dead body cells are eliminated, and new cells are produced.”

Participant number 12 expressed their unfamiliar appearance to others: " Those who have seen me before say very discouraging things like how old you’ve become and your hair has fallen out because they compare me to before, but for those who see me for the first time, it’s normal for them and they don’t say that your skin is ruined. Four months after the raw vegan diet, I had severe detoxification, all the hair on my face fell out, and I got depressed again, I got acne, my blood pressure went up again, I had terrible bone pain that felt like death, detoxification took 10 days, and my hair grew for 5 months” (participant 12).

### On the path of overcoming difficulties

Participants in this study faced multiple challenges during the process of vegetarianism, from physical and psychological changes to social issues and interaction with others. In this regard, they used methods to adapt to this approach and had recommendations for those who chose this path.

#### Associated problems

At the beginning of the participants’ vegetarian journey, they faced problems such as severe hunger, bleeding after menopause, detoxification, social isolation, stress and anxiety, depression, and conflict with the doctor’s recommendations. In this regard, participant number ten remarked: “Physically, I have been menopausal for 5 years, but after 15 days of raw vegetarianism, I had bleeding again for a week, and I thought my menstrual cycles had returned, but it was only for a month, and I think it was my uterine cleansing, and I also had a stomach reflux, and whatever I ate burned my throat, but now I’m fine, and my ears and throat no longer itch.”

Participant number seven, who had a cancerous tumor and a high weight, had started detoxifying his body with the start of vegetarianism, and in this regard, he stated: “My tumor started detoxifying and bad-smelling infections came out of it for 14 months, then it improved, my weight had decreased severely, and I reached 47 kilos.” Participant number 2 also analogized quitting meat to quitting drug addiction.

#### Matching the new way

Participants used various methods to match the new diet. In this approach, eating four categories of foods was forbidden: 1- meat, 2- bread, 3- rice, and 4- dairy. Participants were advised not to use any cooked food if possible and to use foods with live cells (fruits and vegetables, sprouts, grains). Participant number 12 stated: “Another factor that made me continue this path was that I decorated my food, and it was interesting for me, and I took pictures and sent them to raw vegan groups. For the first 6 months, I used to consume fresh lemon every day. … What I mean is that my body used to crave sourness a lot at first, but now it’s adjusted. The body itself demands what it needs more of. I eat very little honey because I believe that natural honey is very scarce and they give sugar and sugar water to the bees, and natural honey that occurs in nature is not available”.

Some participants prepared themselves for vegetarianism with their new interpretations of spiritual meanings. In this regard, Participant Seven expressed his experiences as follows,” Stress makes the blood acidic, and even if you are an absolute raw vegan, you cannot reach peace because stress prevents human peace. We have a cooked food family, but my spouse is a raw vegan, and we are very comfortable. We do not waste our time in front of the gas stove. We wash fruits and vegetables very easily and do not have the problem of cooking food… Now I eat an apple and enjoy it because I see the light of God in it. … I think it’s a waste to hurt myself and inflict the pain I had (before this regimen) and the divine spirit inside me because it’s a shame to harm the body that God gave you with love”.

#### Perceived recommendations

Participants studied various recommendations for those who chose this approach. Some people recommended a gradual start and uttered that people under gradual methods were less physically and mentally pressured. But in some cases, they suggested starting with health and complete vegetarianism. Participant number four stated in this regard: “I recommend that if someone wants to start this method, start by studying. A sick person no longer has the opportunity to start gradually and must start at once, but someone who is not sick or does not have a severe illness should start step by step and diversify into raw vegetarian foods. I made a plant-based cake that looks just like sweet pastry cakes. I even had ear surgery and only used antibiotics for one day and then had herbal teas”.

Participant number 3 also states that the spirit must be improved before starting this diet. Participant number 4 also recommends that after 40 days of following the vegetarian diet, the person becomes accustomed to it and says: “I recommend that you assume experimentally for 40 days that you are on an island with only fruits and vegetables and start raw vegetarianism. Reading is very helpful”.

### Attaining the expected outcomes

Participants had different perceptions of the benefits of a vegan diet. They noticed a healthy lifestyle, improved physical condition, improvement in physical illnesses, improved mental well-being, and a feeling of youthfulness.

#### Healthy lifestyle

Participants made fundamental changes in their lifestyles. They were supporters of the environment and animals. Their diet was rich in water and fruit consumption. Participant number 6 stated: “*Producing one kilogram of meat requires 15,000 liters of water, but less than a thousand liters of water is needed for one kilogram of chickpeas. We have become accustomed to turning on the stove in the morning and then starting our day, which leads to the production of greenhouse gases. Let’s not harm nature and live a natural life*”.

Some participants stated that their ability to exercise has increased with this lifestyle. Participant number 5 stated: “When my weight reached 100 kilograms, I started eating sprouts and anything that could be softened, like soaked chickpeas. I used to take herbal supplements to maintain my energy, but now I get the same energy from eating chickpeas. The human body does not need meat, and whenever I eat chickpeas, it gives me the same energy as meat… I used to go to Mount Taq-Bostan and carry a 30-kilogram backpack on my back and climb the mountain and then run for 2 hours and then eat 10 delicious meat skewers to regain energy, but then I realized that I was wrong and thought that my output was the problem, but I realized that it was not the case, and I only harmed my body and punished it in various ways”. When attending social events, participants brought their own vegetarian food.

#### Therapeutic feedback

Almost all participants experienced weight loss and considered it as a useful point to improve their health status. The clinical symptoms of all the diseases they had were reduced or eliminated in some cases. These diseases included diabetes, cancer, joint arthritis, sexual disorders, varicose veins, and so on. Participant number Six stated: “I had arthritis in my neck and a herniated disc in my back, and as soon as I lost weight, my back disc improved, and sometimes I don’t even feel the neck pain”. Participant number seven has been a raw vegan during pregnancy and describes her experience as follows: “I have given birth to a healthy child without any medication during pregnancy, despite having so many diseases, and now I have brought a healthy child into the world without jaundice, but my two older children had problems at birth”. This diet had improved sleep, and almost all participants mentioned it. Participant number three stated: “I go to bed at 10 pm and wake up at 5 am, a very deep sleep. I used to struggle to sleep with tranquilizers and my eyes closed but my brain was awake”.

#### Enhancing emotional wellbeing

Another desirable effect of the vegetarian diet was the improvement in mood and mental state. These improvements were mentioned with statements such as improved mood, change in temperament, and reduced irritability. Participant number 7 stated: “I believe that cancer patients should not eat cooked food. Whenever I eat cooked food, I become ill-tempered and my pimples come back. Whenever I become ill-tempered, everyone understands I have slipped (eat cooked food). I have found peace with raw veganism and come out with a healthy face.”

Another participant also said: “I used to be aggressive and short-tempered, but since I became a raw vegan, I have found peace and achieved the right way of life, and even if the worst problems come to me, I thank God.” (Second participant). Some individuals also stated that they have undergone a spiritual transformation with this method. In this regard, one of the participants stated: “Every human being is 98% soul and 2% physical body. If the soul heals and becomes aware, the body also heals” (First participant).

#### Feeling of youth

Three participants stated that after starting a vegan diet, their energy has significantly increased and it doesn’t tire them out in their daily and work activities, and they feel a kind of youthfulness. Participant number 3 said: “I have been a raw vegan for 2 years and all my pains have completely gone, my bleeding has stopped completely, my energy has increased, I conquered the peak, I feel young, and I don’t feel like I’m 50s at all… I feel like I’m the same age as my daughters and I don’t feel like I’m 58 at all.”

Another participant mentioned the increase in their energy and said: “After eating fruits, our energy goes up, but with other foods, the body gets tired.” (Participant 11).

## Discussion

This study aimed to explore the experience of people adhering to a vegetarian diet. Before starting the vegetarian path, the participants faced chronic diseases such as diabetes, arthritis, and obesity, and despite multiple visits for treatments, they did not improve and were somewhat desperate. These conditions made people determined to look for another way to improve. Therefore, people turned to vegetarian diets.

Previous studies have shown that vegetarianism may play a role in the treatment of various diseases, including cardiovascular disease, hypertension, diabetes, obesity, and cancer. Given the growing evidence on the health benefits of vegan diets, vegetarian diets may be used as an adjunctive therapy in disease management [27]. On the other hand, it has been observed that raw vegetarianism by focusing on indicators such as body weight, serum fat level, rheumatoid arthritis and fibromyalgia symptoms, tooth erosion, stool microflora, vitamin B12 status, consumption of antioxidants and other nutrients has positive effects and is good for health. However, side effects such as decreased HDL, and amenorrhea have also been reported [28].

Despite the positive effects of vegetarianism on health, removing disappointment, improving quality of life, and spirituality as shown in the present study, contradictory results have been reported in other research. A 2018 study found that vegetarians had lower self-esteem, less meaning in life, and more negative moods compared to semi-vegetarians and omnivores [29]. Additionally, this study showed that psychological adaptations were less in vegetarians than in other groups, a result that has been confirmed by other studies [30]. Nevertheless, mental disorders may come before the acceptance of vegetarianism [29]. These findings may be influenced by contextual social factors, necessitating further investigation through additional studies.

Another study in 2023 also revealed that vegetarians perceived more negative treatment from others compared to semi-vegetarians and omnivores. Additionally, they believed that strangers were more likely to understand this negative behavior [31]. This aligns with the acknowledgment from participants in our study that others hold a negative view towards their choice to be vegetarian and stick to it. Despite the limited research on the impact of vegetarianism on quality of life, one study found that opting for raw vegetarian diets can lead to improved physical health, positive moral attitudes, a greater sense of community belonging, and reduced environmental impact [32].

In this study, the participants had a unique meaning framework towards continuing the path of vegetarianism and considered it as God’s miracle, God’s will, and a spiritual path, and they also tried to raise their awareness continuously by using virtual space and reading books. Various factors can influence the adoption of a vegetarian diet. One of these factors is the ethical concerns related to the killing of animals. Another is the increased health and potential beneficial effects of raw vegetarianism, which leads to an inclination towards it. It has also been observed that religions that encourage the avoidance of meat consumption and concern about the environmental effects of meat production are important motivations for accepting vegetarianism [33]. Therefore, it can be said that following a vegetarian diet can be considered a social identity because it reflects the motivations, feelings, and attitudes of those who choose it [34]. Qualitative research conducted in 2023 revealed that the advantages of vegetarianism are influenced by individuals’ perspectives, principles, and ethical convictions. However, the findings of the study indicated that vegetarians experience better health-related outcomes compared to non-vegetarians [35].

Most of the participants started the vegetarian diet with doubts and hesitation and were repeatedly tempted to return due to various reasons such as family criticism and changes in physical appearance. It can be said that many social and psychological factors affect people’s adherence to vegetarian diets. These effects may support or prevent a healthy lifestyle [36]. A study in Saudi Arabia showed that social norms including common food culture (meat consumption) on special occasions, holidays, and family gatherings, and showing generosity by preparing meat on these occasions have a significant effect on non-adherence to a vegetarian diet [37]. Another research study highlighted the significant impact of social identity on dietary choices and how it shapes individuals’ perspectives on avoiding meat consumption [38]. Financial cost, enjoyment of meat consumption, inadequate cooking skills, and lack of access have been identified as other barriers affecting adherence or non-adherence to vegetarian diets [37].

Starting a vegetarian diet can be challenging due to several factors, including extreme hunger, detoxification process, stress, anxiety, and conflicts with the recommendations of the attending physician. These barriers are complex and interrelated, and they can affect a person from the initial stages of starting vegetarianism, leading them to stop the diet [39]. Some patients find it difficult to achieve a complete vegetarian diet as they believe it requires more perfectionism and endurance. Additionally, many participants in studies experience problems during the adaptation period, which reduces their motivation to continue with the diet [40].

People took a long time to adapt to the new way of eating. Vegan patients were advised to use decoration and variety in their food, gradually adjust their diet, be aware of the possibility of returning symptoms of diseases, and improve their mood. Based on the participants’ experience, adopting a vegetarian diet led to partial or complete improvement of all disease symptoms. They felt young, and energetic, and led a healthy lifestyle. Moreover, this approach had a positive impact on their mental state and mood.

Our study had limitations related to qualitative research, such as generalizability and objectivity of data. Therefore, it is suggested to conduct further quantitative experimental research on vegan individuals.

## Conclusion

This study aimed to explore the experienced compliance with a vegan diet. Before starting this path, participants faced pre-existing conditions such as diabetes, arthritis, and obesity. Therefore, they created a unique semantic framework for continuing their path and considered it a divine miracle, and also tried to constantly increase their awareness through the use of virtual space and reading relevant books. Most participants started the vegan diet with doubt and hesitation and repeatedly had the temptation to return due to various reasons such as family criticism and changes in physical appearance. They also faced multiple problems such as severe hunger, detoxification process, stress and anxiety, and the conflict of this regimen with the recommendations of the treating physician, and it took a long time for individuals to adapt to the new dietary approach. In this way, recommendations such as using decoration and variety in foods, thinking about the return of disease symptoms, gradually starting the path, improving mood, and adjusting their mood were important. Ultimately, the vegan diet led to relative and complete improvement in all disease symptoms. They had a healthy lifestyle and felt youthful and energetic. Also, this method had a positive impact on the mental and emotional state of individuals. In light of the variances observed in prior studies and the accompanying controversies, it is recommended that future research endeavors employ meticulous quantitative and experimental methodologies to attain comprehensive insights into vegan diets.

## Data Availability

The data will be available upon request to the corresponding author.

## Abbreviations

KUMS: Kermanshah University of Medical Sciences
